# Proliferative and Invasive Colorectal Tumors in Pet Dogs Provide Unique Insights into Human Colorectal Cancer

**DOI:** 10.3390/cancers10090330

**Published:** 2018-09-14

**Authors:** Jin Wang, Tianfang Wang, Yanfang Sun, Yuan Feng, William C. Kisseberth, Carolyn J. Henry, Irene Mok, Susan E. Lana, Kevin Dobbin, Nicole Northrup, Elizabeth W. Howerth, Shaying Zhao

**Affiliations:** 1 Department of Biochemistry and Molecular Biology, Institute of Bioinformatics, University of Georgia, Athens, GA 30602, USA; jw16567@uga.edu (J.W.); tw71066@uga.edu (T.W.); athenaliu0109@gmail.com (Y.S.); yf94402@uga.edu (Y.F.); 2 Department of Veterinary Clinical Sciences, the Ohio State University College of Veterinary Medicine, Columbus, OH 43210, USA; kisseberth.2@osu.edu; 3 College of Veterinary Medicine, University of Missouri, Columbia, MO 65211, USA; HenryC@missouri.edu; 4 Flint Animal Cancer Center, Colorado State University, Fort Collins, CO 80525, USA; Irene.Mok@ColoState.EDU (I.M.); Susan.Lana@ColoState.EDU (S.E.L.); 5 Department of Biostatistics, University of Georgia, Athens, GA 30602, USA; dobbinke@uga.edu; 6 College of Veterinary Medicine, University of Georgia, Athens, GA 30602, USA; northrup@uga.edu (N.N.); howerth@uga.edu (E.W.H.)

**Keywords:** spontaneous canine colorectal tumors, human-dog comparison, cancer cell proliferation and gene mutations, cancer cell invasion and stromal activation, CMS4 and crypt-like or EMT invasion

## Abstract

Spontaneous tumors in pet dogs represent a valuable but undercharacterized cancer model. To better use this resource, we performed an initial global comparison between proliferative and invasive colorectal tumors from 20 canine cases, and evaluated their molecular homology to human colorectal cancer (CRC). First, proliferative canine tumors harbor overactivated WNT/β-catenin pathways and recurrent CTNNB1 (β-catenin) mutations S45F/P, D32Y and G34E. Invasive canine tumors harbor prominent fibroblast proliferation and overactivated stroma. Both groups have recurrent TP53 mutations. We observed three invasion patterns in canine tumors: collective, crypt-like and epithelial–mesenchymal transition (EMT). We detected enriched *Helicobacter bilis* and *Alistipes finegoldii* in proliferative and crypt-like tumors, but depleted mucosa-microbes in the EMT tumor. Second, guided by our canine findings, we classified 79% of 478 human colon cancers from The Cancer Genome Atlas into four subtypes: primarily proliferative, or with collective, crypt-like or EMT invasion features. Their molecular characteristics match those of canine tumors. We showed that consensus molecular subtype 4 (mesenchymal) of human CRC should be further divided into EMT and crypt-like subtypes, which differ in TGF-β activation and mucosa-microbe content. Our canine tumors share the same pathogenic pathway as human CRCs. Dog-human integration identifies three CRC invasion patterns and improves CRC subtyping.

## 1. Introduction

Spontaneous canine cancers represent one of the best animal models of human cancers [1,2,3,4,5,6,7,8,9,10,11]. Being naturally occurring, heterogeneous and with an intact immune system, they capture the essence of human cancer, unlike most genetically modified or xenograft rodent models. Furthermore, dogs better resemble humans in biology, e.g., similar telomere and telomerase activities [12] and more frequent spontaneous cancers of epithelial origin [2,8,9,10,11], unlike mice [13]. Notably, dogs share the same environment as humans and hence are exposed to the same carcinogens. Indeed, similar risk factors for cancer development (advancing age, obesity, diet, etc.) and numerous dog-human anatomic/clinical homologies for the same type of cancer have been noted [1,2,3,11].

Unlike human cancers where hundreds of thousands of cancer cases have been characterized with genome-wide approaches [14,15,16,17,18,19,20,21], far fewer canine cancers have been studied. As a result, we have a limited molecular understanding of canine cancers, which makes this immensely valuable resource significantly understudied and underused.

With 140,250 new cases and 50,630 deaths estimated in 2018 [22], colorectal cancer (CRC) is the third most common cancer in the US. Thus, to better understand and treat CRC is important. We have previously characterized copy number abnormalities (CNAs) in canine CRC genomes [10], which supports the dog-human molecular homology. Furthermore, we have successfully developed a novel dog-human comparison strategy for cancer driver-passenger discrimination for amplified/deleted genes [7,23].

To further understand colorectal carcinogenesis mechanisms in pet dogs and their homology/difference with their human counterparts, we set out to investigate gene expression alteration, mutations, and microbiota changes of intestinal tumors from 22 pet dogs, as described below.

## 2. Results

### 2.1. RNA-Seq Analysis Clusters the Tumors into Two Major Groups

**Figure 1:**
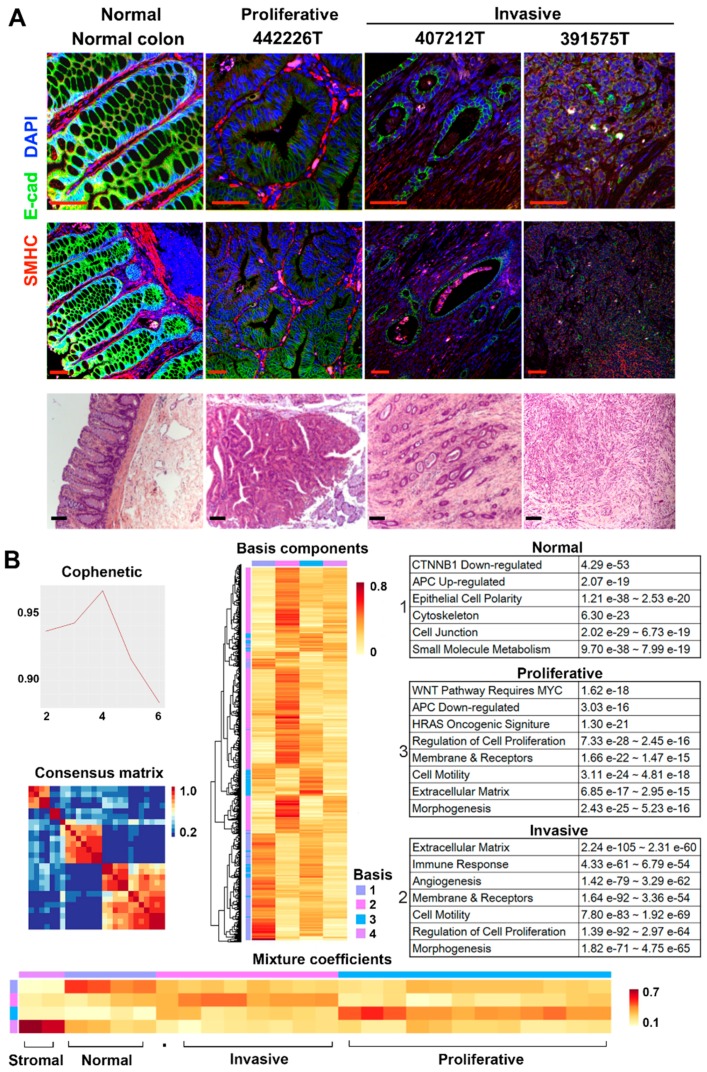
RNA-seq analysis clusters the tumors into two major groups-proliferative or invasive. (**A**) Representative confocal (top two panels) and H&E staining (bottom panel) images of canine colon normal tissues, proliferative and invasive tumors. E-cad: E-cadherin; SMHC: smooth muscle myosin heavy chain (SMHC). Scale bar: 100 μM. (**B**) Non-negative matrix factorization (NMF) clustering identifies four metagenes (top left and middle columns), three of which have significantly enriched functions (top right column). The metagenes cluster the samples into four groups (bottom), with invasive and proliferative ones being the largest. See also Table S1 and Figure S1.

We performed RNA-seq on 26 intestinal samples collected from dogs with spontaneous tumors in the large intestine (20 dogs) and the small intestine (two dogs), and without any intestinal tumors detected (one dog) (Table S1). Among the samples, 23 are tumors consisting of colorectal adenomas from four dogs, adenocarcinomas (12 colorectal and one each for duodenum and jejunum) from 17 dogs, and two colonic stromal tumors from one dog (Table S1). Three samples are normal colonic epithelial tissues from two canine patients described above and one normal dog. Histologically, the 4 adenomas and 17 adenocarcinomas can be largely classified into two groups: highly proliferative (4 adenomas and 9 adenocarcinomas) or highly invasive (8 tumors). Highly proliferative tumors are characterized by prominent proliferation of epithelial cells that are clearly marked by E-cadherin staining (Figure 1A). Highly invasive tumors are characterized by: (1) the spread of tumor cells into submucosa and muscle layers of the intestine; and (2) the lack of prominent proliferation of clearly marked epithelial cells (Figure 1A).

Some of the tumors clearly have more stromal cell content (Figure 1A). To reduce variations, we maximally dissected away stromal regions without any tumor or epithelial cells, and only used sections enriched in tumor or epithelial cells for RNA-seq and other genomic analyses. We performed RNA-seq analysis with digested tissues of the 26 samples (Table S1A).

We then conducted non-negative matrix factorization (NMF) [24] clustering analysis with 10,618 total genes that are expressed in at least one sample (Table S1B). The analysis identified four metagene sets and four sample clusters (Figure 1B). First, the two stromal tumors form one NMF cluster, and the three normal samples and one tumor constitute another (Figure 1B). The remaining two clusters nicely separate highly proliferative tumors from highly invasive tumors: one cluster consisting of 12 (out of 13 total) proliferative tumors, while the other containing 7 (out of 8 total) invasive tumors and one proliferative tumor (Figure 1B). Thus, the results are consistent with the histopathological classification as illustrated in Figure 1A. Metagenes identified are also informative. Metagenes of the normal group are significantly enriched in functions that characterize differentiated colon epithelial cells (Figure 1B, Table S1C). These include β-catenin-downregulated targets, APC-upregulated targets, epithelial cell polarity and others. The opposite was noted for metagenes of the proliferative group. Metagenes of the invasive group are significantly enriched in features of cell invasion, e.g., extracellular matrix, etc. (Figure 1B).

NMF clusters are supported by unsupervised hierarchical clustering with various numbers of top most variable genes in expression across the 26 samples. The analysis consistently separates invasive tumors from proliferative tumors (Figure S1). The same is achieved with the principle component analysis (PCA) with the entire transcriptome.

In summary, histopathological and three gene expression clustering strategies have consistently classified the tumors into two major groups, highly proliferative or highly invasive. Below are our molecular characterizations of each group.

### 2.2. Canonical CRC Pathways Are Activated in Proliferative Tumors

**Figure 2:**
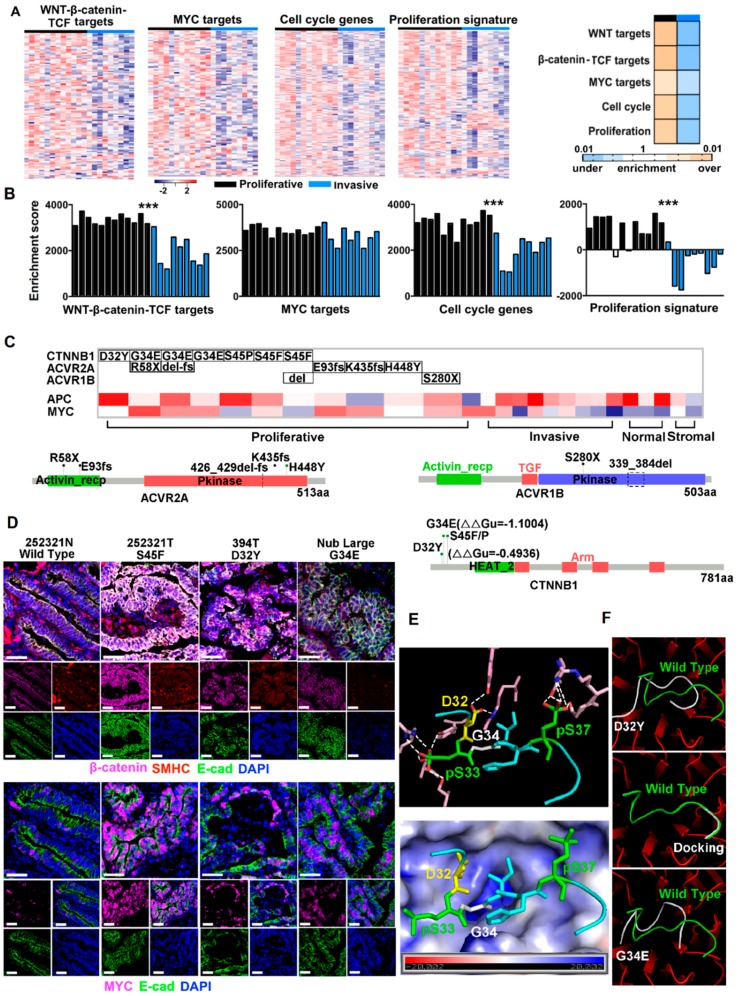
Proliferative tumors harbor activated WNT-β-catenin cell proliferation pathways and underlying gene mutations. (**A**) Left four heatmaps indicate higher expression levels, represented by $\log_{2}(FPKM)$, of gene signatures shown in proliferative tumors than invasive tumors, with their GSEA p-values specified by the right heatmap. (**B**) The bar plots indicate ssGSEA enrichment scores of the same gene signatures, reaching the same conclusion as (A), ***: *p* < 0.001. (**C**) Proliferative tumors harbor recurrent mutations of CTNNB1, ACVR2A and ACVR1B, as well as recurrent *APC* downregulation and *MYC* upregulation. Mutations of the three genes are shown at the bottom with their protein domains indicated. ∆∆Gu, estimated as previously described [11], predicts if a missense mutation will alter the protein 3D structure. (**D**) Representative IHC images indicate the enrichment of cellular and nuclear β-catenin (top) and nuclear MYC (bottom) in tumor cells harboring CTNNB1 mutations. (**E**) The top 3D structure indicates that the phosphorylated N-terminal peptide of β-catenin binds β-TrCP1 through hydrogen bonds (dashed white lines) via D32, pS33 and pS37 of β-catenin. The bottom 3D structure indicates that the binding site locates in a positively charged pocket formed by β-TrCP1, and G34 of β-catenin locates at the center of the pocket. (**F**) Docking of β-catenin peptides to β-TrCP1 indicates that D32Y and G34E mutations alter substrate binding. The ground truth peptide binding in the crystal structure [37] is shown green, while peptide docking is shown in white. See also Figure S2, Table S2.

To better understand the difference between proliferative and invasive tumors identified in Figure 1, we performed gene set enrichment analysis (GSEA) with signature gene groups used in subtyping [25,26,27,28,29,30] and characterization [31,32,33,34,35] of human CRCs. These include 17 canonical CRC signatures, 11 cancer pathways, 9 stromal signatures, 33 specific immune processes and 14 specific metabolic processes, totaling to 3881 genes (Table S2A,B). The analysis reveals that activation of WNT/β-catenin signaling → cell cycle and proliferation is the most significant feature of proliferative tumors (Figure S2A, Table S2A). Indeed, intestinal WNT/β-catenin/TCF signature [33], crypt proliferation signature [34] and cell cycle activation signature [25] are all significantly (*p* < 0.05) enriched in proliferative tumors (Figure 2A). Although not as significant, MYC targets are also upregulated in proliferative tumors (Figure 2A). These results are consistent with single sample GSEA (ssGSEA) (Figure 2B). In summary, these canine proliferative tumors share similar molecular features as canonical CRCs in humans [14,15,16,18,19,25].

### 2.3. CTNNB1 and TGF-β Signaling Genes Were Recurrently Mutated in Proliferative Tumors

To understand the mechanisms underlying the observed WNT/β-catenin → cell proliferation activation (Figure 2A,B), we performed Whole Genome Sequencing (WGS) for 15 tumor and matching normal samples of 10 canine colorectal tumor cases (Table S1). We then combined WGS and RNA-seq data (Table S1A), which significantly increases the sequence coverage for mutation finding.

*CTNNB1*, which encodes β-catenin, is the most noteworthy. It is mutated in 7 (50%) proliferative tumors. Importantly, the mutations are S45P, S45F, G34E and D32Y (Figure 2C, Table S2C). All locate in the N-terminal peptide of D(32)S(33)G(34)IHSGATTTAPS(45)LS of β-catenin, where phosphorylation of the Ser/Thr residues initiates β-catenin degradation [36]. These mutations are likely gain of function and colorectal tumorigenesis drivers. First, S45P/F mutations would prevent S45 phosphorylation and hinder β-catenin degradation. Indeed, our IHC experiment reveals increased accumulation of β-catenin inside tumor cells that harbor S45F mutation (Figure 2D). Furthermore, these tumor cells also express substantially more MYC protein in their nucleus (Figure 2D).

Following N-terminal Ser/Thr phosphorylation, β-catenin is targeted for ubiquitination and degradation. D32Y and G34E mutations are likely to affect this process, as “D(32)pS(33)G(34)IHpS” marks the “DpSG*φ*XpS” destruction motif [37]. To better understand this, we studied the crystal structure of human β-TrCP1/Skp1/β-catenin [37], an E3 ligase complex that ubiquitinates β-catenin (note that except for a T60S change, canine β-catenin is identical to human β-catenin; see Figure S2B). The N-terminal phosphorylated peptide of β-catenin binds β-TrCP1 via hydrogen bonds and electrostatic interactions [37] (Figure 2E and Figure S2C), some of which would be disrupted by the D32Y mutation. Likewise, G34 locates in a positively charged environment [37] (Figure 2E), and the G34E mutation would change the electrostatic interaction. Indeed, our substrate docking modeling indicates that both mutations alter the binding of β-catenin to β-TrCP1 (Figure 2F). Our IHC experiment reveals substantial accumulation of β-catenin and MYC in tumor cells with D32Y and G34E mutations (Figure 2D).

Even though we did not find notable *APC* mutations, *APC* is recurrently downregulated, especially in proliferative tumors (Table S2D). Its lowest expression level was observed in a proliferative tumor (Figure 2C) that harbors neither *CTNNB1* mutations nor mutations described below.

Besides *CTNNB1*, we also uncovered mutations in *ACVR2A* and *ACVR1B*, which encode receptors of activin, a member of the TGF-β superfamily, currently in proliferative tumors (Figure 2C).

### 2.4. Cancer-Associated Fibroblast (CAF) and Stromal Signatures Are Activated in Invasive Tumors

**Figure 3:**
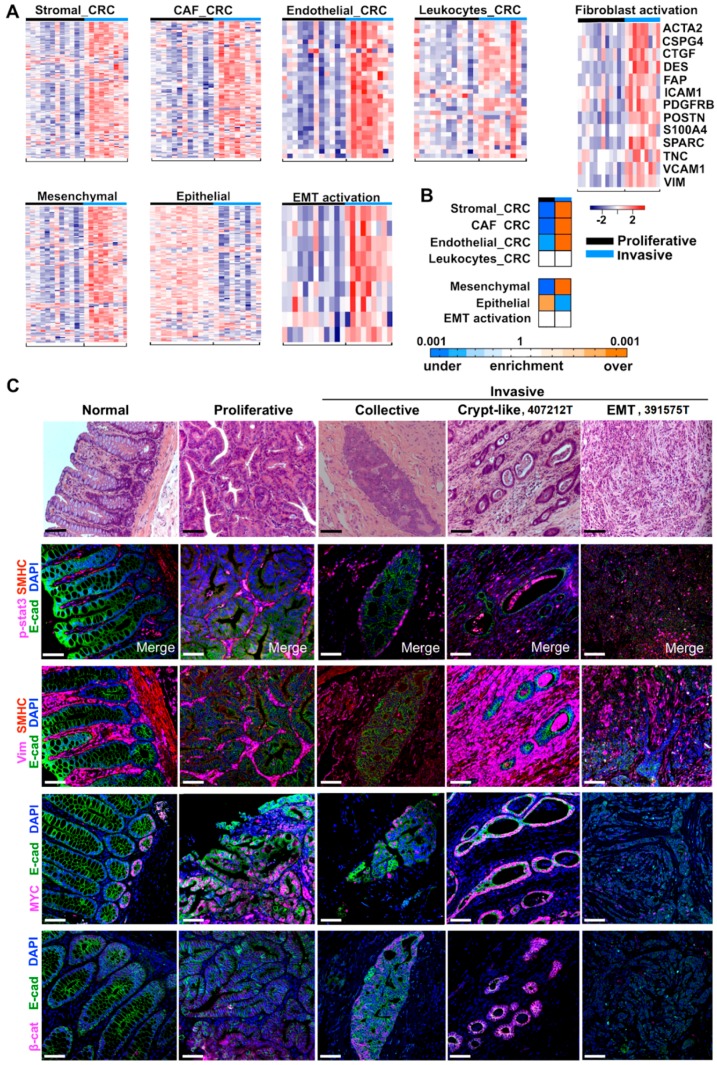
Stromal signatures are activated in invasive tumors and three invasion modes are observed. (**A**) Heatmaps indicate higher expression levels of stromal and EMT signature genes in invasive tumors than in proliferative tumors. Heatmaps are presented as described for Figure 2A. (**B**) Heatmaps of the GSEA p-values of the signatures indicated. (**C**) Representative IHC images of normal colon, proliferative tumor, and tumors of three invasion modes. In crypt-like invasion (407212T), tracks of MYC-positive crypt-like structures are surrounded by dense and multilayers of fibroblasts. β-cat: β-catenin. See also Figure S3, Tables S2 and S3.

Besides canonical CRC pathways (Figure 2A), we also investigated tumor microenvironment. We found that stromal signatures derived from human CRC [32] are activated in canine invasive tumors, compared to proliferative tumors. Specifically, CAF and endothelial cell signatures are significantly enriched, while the leukocyte signature is not (Figure 3A,B and Table S2A,B). Interestingly, similar conclusions were reached with stromal signatures derived from single cell RNA-seq analysis of human melanoma [38]. CAF, macrophage and endothelial cell signatures are enriched in invasive tumors, whereas signatures of B-cells and T-cells are not (Figure S3A,B). Lastly, consistent with CAF signature enrichment, fibroblast activation markers are upregulated in invasive tumors (Figure 3A, Table S3).

Epithelial–mesenchymal transition (EMT) has been extensively studied in human CRC. To investigate EMT in these canine tumors, we examined its signatures from human CRC [26,35]. As expected, the epithelial signature is significantly enriched in proliferative tumors, whereas the mesenchymal signature is significantly enriched in invasive tumors (Figure 3A,B). The EMT activation signature is upregulated in invasive tumors, albeit not as significantly (Figure 3A,B).

### 2.5. Three Modes of Cancer Cell Invasion Were Observed

Both EMT and CAF signatures are upregulated in invasive tumors (Figure 3A,B). To better understand this, we performed IHC experiments with vimentin, a mesenchymal cell marker frequently used for fibroblast and CAF identification, as well as E-cadherin, an epithelial marker. The result supports the gene signature analysis shown in Figure 3A,B. Fibroblast proliferation is clearly more prominent in invasive tumors than in proliferative tumors, while no difference was found for pSTAT3 (Figure 3C), a marker often associated with immune response.

Importantly, the IHC study reveals three modes of tumor cell invasion: collective, crypt-like and EMT (Figure 3C). Collective and EMT invasions are both well studied in human cancers [39,40]. We observed collective invasion in canine proliferative tumors, with masses that consist of predominantly epithelial cells, with far fewer fibroblasts, found in submucosa and muscularis layers of the colon (Figure 3C). We also observed EMT in canine invasive tumors, with numerous tumor cells expressing E-cadherin and vimentin simultaneously (Figure 3C).

“Crypt-like” is another invasion mode frequently observed in our canine invasive tumors. In this mode, crypt-like structures, consisting of a monolayer of epithelial cells that are surrounded by densely populated and multilayered fibroblasts, were found in submucosa and muscularis layers of the colon (Figure 3C and Figure S3C). Crypt-like invasion differs from collective invasion in: (1) no significant epithelial cell proliferation (monolayer versus multilayer); and (2) very prominent fibroblast proliferation. Crypt-like invasion also differs from EMT invasion, as epithelial cells and mesenchymal cells are easily distinguishable. 

Crypt-like invasion is not as extensively reported as collective or EMT invasion; we hence use an invasive cancer, 407212T, as an example for further illustration. Tumor cells of 407212T have penetrated through the colon and have likely metastasized to the lung (Table S1). Our IHC staining reveals clear tracks of crypt-like structures (Figure 3C and Figure S3C), as if tumor cells have been walking through the colon. Crypt-like structures vary considerably in size. Each has a monolayer of epithelial cells with distinct cell-cell junction, as indicated by E-cadherin and β-catenin staining (Figure 3C), resembling colonic crypts. However, they also differ from normal crypts. First, not just surrounded by extensive fibroblasts, many crypt-like structures harbor fibroblasts inside their lumen (Figure 3C). Second, their epithelial cells are nearly all MYC-positive, matching crypt stem cells or progenitors but not fully differentiated cells (Figure 3C). This is supported by their activated signatures of hypoxia and cellular response to oxidative stress (Figure S3D). Thus, these crypt-like structures consist of cells with colon stem cell or progenitor features.

### 2.6. Crypt-Like Invasion Tumor Harbors Mucosa-Like Microbiome

**Figure 4:**
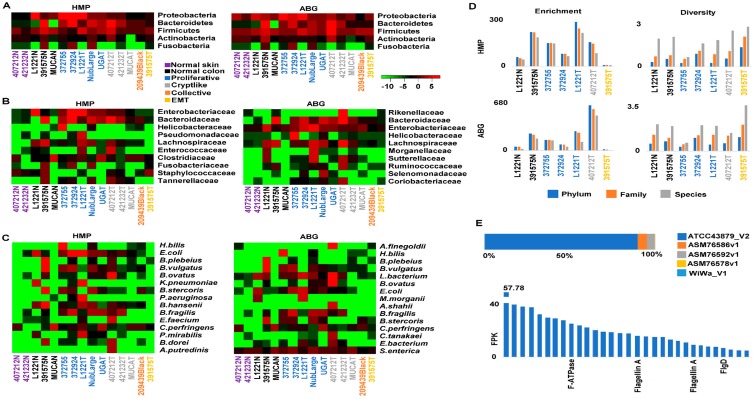
A crypt-like invasion tumor harbors a microbiome that resembles mucosa samples. (**A**–**C**) Heatmaps indicate enrichment levels (Red: enriched; green: depleted) of bacterial phylum, family and species in each sample. HMP (Human Microbiome Project) and ABG (all bacterial genomic sequences) are the two microbial databases used. Sample types are specified by the colors as indicated. (**D**) Crypt-like invasion tumor 407212T (gray), but not EMT invasion tumor 391575T (yellow), resembles normal colorectal mucosal samples (black) and proliferative tumors (blue) in bacterial enrichment and diversity. (**E**) *H. bilis* strain ATCC 43879 is enriched in proliferative tumor 372755 (top) and expresses genes including those encoding flagellin A (bottom). See also Table S4.

Gut microbiome has gained increasing attention in human CRC research. For an initial understanding of the microbiomes of our canine tumors, we searched for microbial sequences in their WGS data (Table S1A), as previously described [11]. As expected, canine colorectal samples contain >100-fold more bacterial sequences than skin samples (Figure 4A–C, Table S4A–C). Importantly, these colorectal samples are enriched in three bacterial phyla: bacteroidetes, proteobacteria and firmicutes (Figure 4A, Table S4A). This is supported at the family level, where the top enriched families include bacteroidaceae, enterobacteriaceae, rikenellaceae and helicobacteraceae (Figure 4B, Table S4B). At the species level, top abundant bacteria also belong to these three phyla, although the actual species vary in each sample (Figure 4C, Table S4C). One difference between our findings and published human and canine colon microbiota data [41,42] is that proteobacteria, but not fusobacteria, is among the top 3 most enriched phyla.

Tumor 407212T, which exemplifies crypt-like invasion (Figure 3C), is especially noteworthy. Although located in the muscularis layers of the colon and distant from the mucosa, this tumor harbors a microbiome with enrichment and diversity values as high as those of mucosa samples, including normal tissues and proliferative tumors (Figure 4D, Table S4D). One species, *Alistipes finegoldii*, a commensal gut microbe and belonging to the phylum of bacteroidetes, is abnormally enriched (Figure 4C). Please note that *A. finegoldii* has been detected in blood samples of human CRC patients [43]. On the contrary, the EMT tumor (391575T; see Figure 3C) is significantly depleted in bacteria (Figure 4D).

*Helicobacter bilis* has been linked to inflammatory bowel disease (IBD) and CRC in mouse models [44]. We noted that *H. bilis* is significantly enriched in a proliferative tumor (372755T) (Figure 4C). Among its strains examined, ATCC43879 is >16-fold more enriched than others, with its top expressed genes encoding flagellin A and others (Figure 4E, Table S4E). 

### 2.7. TP53 Is Recurrently Altered in Both Proliferative and Invasive Tumors

**Figure 5:**
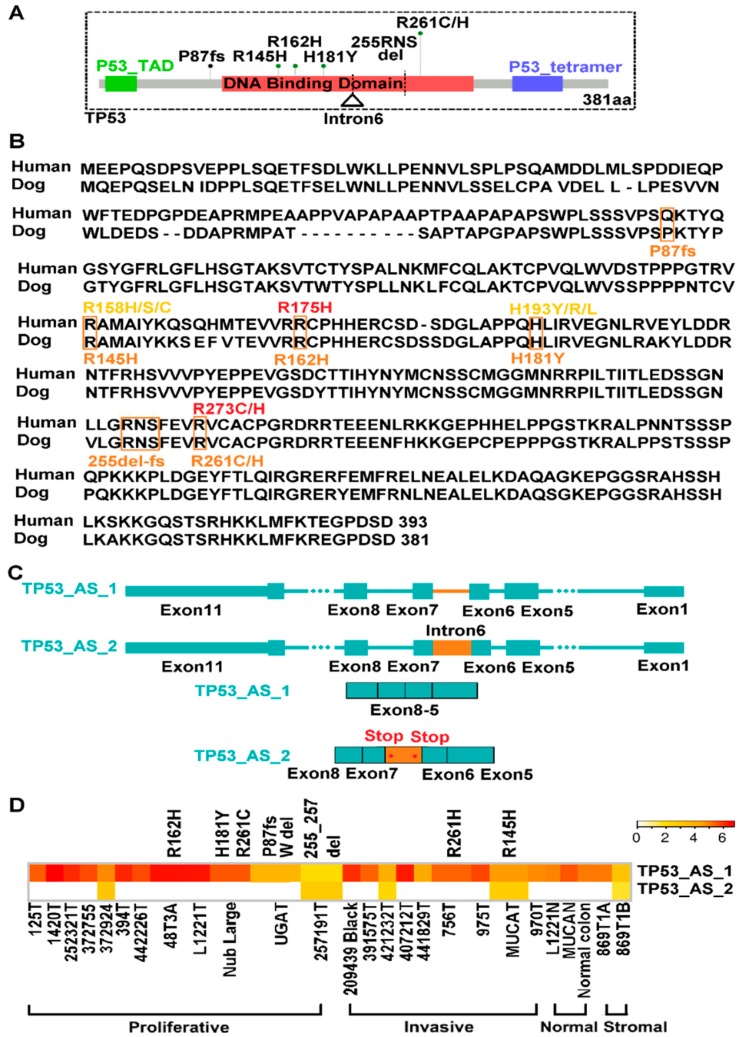
TP53 is recurrently altered in both proliferative and invasive tumors, with some being known drivers. (**A**) TP53 mutations include whole gene deletion, indicated by the dashed lines, and other changes shown. (**B**) Human and dog TP53 protein alignment, with canine mutations and some of their human counterparts (e.g., R175H and R273C/H) indicated below and above the alignment respectively. (**C**) Intron 6 retention, yielding two premature stop codons, was detected. (**D**) TP53 is altered in both proliferative and invasive tumors. The heatmap indicates the abundance of the two transcripts shown in (C). See also Table S5 and Figure S4.

Unlike *CTNNB1* and *ACVR2A/1B* (Figure 2C), we detected TP53 mutations (whole gene deletion, indels and missense mutations) in both proliferative and invasive tumors (Figure 5A,D and Table S5A). Missense mutations identified are all located in the DNA binding domain and are also common in human cancer. For example, through protein alignment (Figure 5B, Table S5B), canine R162H and R261C/H are equivalent to human R175H and R273C/H, respectively. Both are among the top three most frequent TP53 mutations in human CRC (Figure S4) and are known cancer drivers [45].

Finally, we observed intron 6 retention in a fraction of *TP53* transcripts in both proliferative and invasive tumors (Figure 5C,D). Intron 6-retention will create two stop codons within the TP53 DNA binding domain (Figure 5A,C).

### 2.8. We Identified Three Types of Invasion in Human Colon Cancers

**Figure 6:**
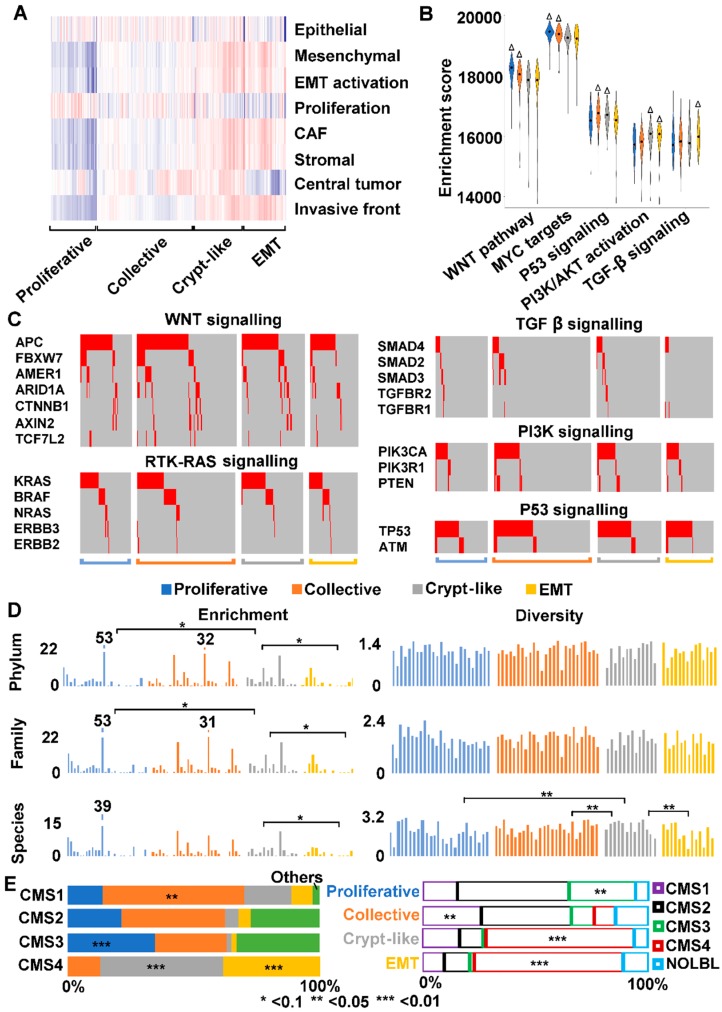
We have classified TCGA colon cancers and found that consensus molecular subtype 4 (CMS4) consists of crypt-like and EMT invasion cancers. (**A**) TCGA colon cancers (379 out of 478 total) were classified into four subtypes, with their ssGSEA enrichment scores of signatures indicated by the heatmap. Red: enriched; blue: depleted. (**B**) The four subtypes differ in canonical CRC pathways, as indicated by the distribution of ssGSEA enrichment scores represented by the violin plot. ∆: significant activation. (**C**) TGF-β signaling genes are less frequently mutated in EMT invasion compared to other subtypes. Each column represents a tumor sample, and only driver mutations (red) are shown. (**D**) EMT tumors overall harbor fewer bacteria, compared to tumors of other subtypes. (**E**) CMS4 can be further divided into crypt-like invasion and EMT invasion subtypes. Left bars indicate the distribution of our subtypes among the CMS subtypes, while right bars indicate the opposite. Others: not classified by us; NOLBL: no label (not classified [25]). See also Figure S5 and Table S6.

To further evaluate the dog-human molecular homology, we tried to identify the four molecular subtypes illustrated in Figure 3C (i.e., proliferative and three types of invasion: collective, crypt-like and EMT) among the 478 human colon cancers from The Cancer Genome Atlas (TCGA) [16]. Guided by our canine findings (Figure S5A), we studied the distribution and clustering of ssGSEA enrichment scores of CRC signatures of: (1) proliferation [34]; (2) EMT (epithelial, mesenchymal, and EMT activation) [26,35]; (3) CAF and stroma [32]; and (4) central tumor and invasive front [30] (Figure S5B). We also included developmental signatures on: (1) colonic stem cells, progenitors and differentiated cells [34]; and (2) colon crypt and top [31]. We identified 74 proliferative tumors, 159 tumors of collective invasion, 79 tumors of crypt-like invasion, and 67 tumors of EMT invasion (Table S6A). These total 379 tumors, accounting for 79% of all TCGA colon cancers examined. Proliferative tumors show the largest differences from other tumors in all CRC signatures examined except for epithelial signature (Figure 6A). Among the three invasive subtypes, collective invasion displays more suppressed signatures related to stroma (mesenchymal, EMT activation, CAF, stromal and invasive front) (Figure 6A). Finally, crypt-like and EMT invasions are similar, except that the former has more activated signatures of central tumor and cell proliferation (Figure 6A).

The four subtypes differ significantly in several aspects in canonical CRC pathway alterations and gene mutations. First, proliferative and collective invasion subtypes both harbor more activated WNT pathway and MYC targets, but more suppressed PI3K/AKT signaling (Figure 6B, Table S6B). Second, crypt-like invasion and EMT invasion both have a higher mutation rate of TP53 (Figure 6C and Figure S5C, Table S6C). Yet, TP53 signaling is enhanced in crypt-like invasion (Figure 6B). Third, EMT invasion harbors the most activated TGF-β signaling and overall the fewest mutations in relevant genes, a clear difference from other subtypes (Figure 6B,C and Figure S5C, Table S6B,C).

We also investigated the difference in microbiome among the four subtypes. First, we identified WGS data from TCGA that are available to 51 proliferative tumors, 98 tumors of collective invasion, 29 tumors of crypt-like invasion and 31 tumors of EMT invasion (Table S6D). Then, we performed the same analysis as described for canine tumors (Figure 5). We noted that crypt-like invasion tumors have similar or even higher bacterial enrichment and diversity, when compared to proliferative and collective invasion tumors (Figure 6D, Table S6D). EMT invasion tumors, however, consistently harbor fewer bacteria (Figure 6D, Table S6D). The observations agree with our canine findings (Figure 4D).

### 2.9. We Classified Consensus Molecular Subtype 4 (CMS4) into Crypt-Like and EMT Invasions

We examined the relationship between our subtypes and the four CRC consensus molecular subtypes (CMSs) from a well-cited study [25]. A total of 419 TCGA colon cancers were investigated by both methods (Table S6E). We noted a significant overlap (>50%) between our collective invasion and CMS1 (Figure 6E, Table S6E). CMS1 also harbors smaller fractions of proliferative, crypt-like and EMT subtypes of ours. CMS1 is characterized by hypermutation, microsatellite instability, and strong immune activation [25]. We also observed a significant overlap between our proliferative subtype and CMS3 (Figure 6E, Table S6E). CMS3 also contains collective and unclassified colon cancers by us (Figure S5, Table S6E). CMS3 is epithelial and has evident metabolic dysregulation [25]. CMS2 is also epithelial and is characterized with WNT and MYC signaling activation (thus the canonical subtype) [25]. It consists of our proliferative and collective invasion subtypes and cancers that are not classified by us (Figure 6E, Table S6E), none of which is enriched. The most interesting finding, however, is that our crypt-like and EMT invasion subtypes are both highly enriched in CMS4, accounting for 87% of all CMS4 tumors (Figure 6E, Table S6E). CMS4, being mesenchymal and with stromal invasion, is featured with prominent TGF-β activation [25]. Yet, our study further classified CMS4 into EMT invasion and crypt-like invasion, with TGF-β activation found only in the EMT invasion subtype (Figure 6B).

## 3. Discussion

### 3.1. Canine Colorectal Tumors Follow Canonical Pathogenic Pathways of Human CRC

Alteration of WNT signaling pathway [46], observed in >90% human CRCs [16], leads to MYC activation, cell proliferation and ultimately tumorigenesis [16]. We have reached the same conclusion for proliferative colorectal tumors in dogs. One interesting difference lies in *CTNNB1*, which is mutated in <10% of human CRCs [16] but in >60% of our canine proliferative tumors. Please note that *CTNNB1* mutations detected in our canine tumors are S45P/F, D32Y and G34D, which interfere with β-catenin ubiquitination and degradation, yielding the same outcome as *APC* mutation. Intriguingly, we did not find frequent *APC* mutation in these canine samples, unlike human CRC [16], although we noted recurrent downregulation of *APC*. We do not know if this is related to the local genomic environment of *APC*. While canine *APC* locates at the chromosome end (near heterochromatin), human *APC* lies in the middle of chromosome 5 (euchromatin). Future study with a larger sample size is clearly required to answer the question. We nonetheless emphasize that whether it is *APC* mutation or *CTNNB1* mutation, the outcome remains the same-activation of WNT signaling.

Alteration of TGF-β signaling pathway also leads to MYC activation and cell proliferation in human CRC [16]. Analogous to human CRC [16], we found recurrent mutation in TGF-β signaling genes *ACVR2A* and *ACVR1B* in our canine proliferative tumors.

Alteration of TP53 pathway occurs in more than half of human CRCs [16]. Comparable to this, *TP53* is recurrently mutated in our canine tumors. Moreover, most mutations detected have been reported in human CRC, with some already classified as drivers [45]. Please note that *TP53* mutations are found in both proliferative and invasive canine tumors, unlike *CTNNB1*. This is consistent with the Vogelstein model that places *TP53* mutation at a later carcinogenesis stage of human CRC [47]. Lastly, we have detected a stop-codon-creating intron-retention in canine tumors. More studies are needed to determine if a truncated TP53 protein is indeed generated and, more importantly, how this has happened. For example, is it due to mis-splicing, and/or because nonsense-mediated mRNA decay is off or dysfunctional?

### 3.2. We Have Detected Three Invasion Modes of Canine Cancer Cells

Microenvironment is important in cancer development and invasion [48,49]. Stromal signatures reported for human CRC [32] are activated in our invasive canine tumors, supporting the dog-human molecular homology. Importantly, we have detected three modes of cancer cell invasion in our canine tumors: collective, crypt-like and EMT. Collective and EMT invasions are both well studied in human cancers [39,40]. Collective invasion is largely defined as migration of a group of cells while maintaining cell-cell contacts. These cells are often epithelial in nature and thus can be readily distinguished from the microenvironment. This is unlike EMT invasion, where many cancer cells have acquired stromal cell features.

To our knowledge, crypt-like invasion, where cancer cells spread via crypt-like structures, is not as extensively reported as collective or EMT invasion. Our study indicates that these cancer cells are MYC-positive, resembling crypt stem cells or progenitors. We propose that they are capable of crypt development in non-mucosa locations because of prominent fibroblast proliferation, which has remodeled the microenvironment to be more mucosa-like (supported by their microbiota that resembles mucosa samples). Whether this is true and how this occurs of course need more research. For example, the origin of the proliferating fibroblasts is unclear. Are they derived from some types of crypt mesenchymal stem cells that migrate with the cancer cells? Or are they local?

### 3.3. Human CMS4 Colon Cancers Consist of Crypt-Like and EMT Invasion Subtypes that Differ in TGF-β Signaling

Most human CRCs can be classified as one of the four consensus molecular subtypes (CMS1, CMS2, CMS3 and CMS4), each with distinct molecular features [25]. CMS4 is the “mesenchymal” subtype, characterized with TGF-β activation, stromal invasion and angiogenesis [25]. Our analysis indicates that CMS4 actually consists of two subtypes, EMT and crypt-like invasion. Although EMT and crypt-like invasions are indeed very similar molecularly, our analysis reveals a few differences. First, only EMT invasion harbors TGF-β activation, likely due to less frequent mutation of TGF-β signaling genes. Crypt-like invasion, meanwhile, displays more activated signature of central tumor and, as discussed previously, may harbor some types of stem cells [15]. We plan to validate this finding using a large sample size, including rectum cancers, in the future. In addition, we plan to include more signatures and parameters, including the consensus Immunoscore calculated based on the density of CD3^+^ and CD8^+^ T-cells within central tumor and invasive front from a recent publication [50].

Microbiome could represent another difference. In crypt-like invasion, the tumors appear to retain the mucosa microbiota after spreading to foreign locations. In EMT invasion, however, the tumors seem to have lost the mucosa microbiota. A recent publication [51] reports that *Fusobacterium nucleatum* and other microorganisms of human colorectal tumors are retained in metastatic sites, and that antibiotic treatment inhibits tumor growth in mouse models. Thus, it would be useful to perform deeper microbiome comparison between EMT and crypt-like invasions, including metagenomics data from stool samples.

Although more studies are needed, our findings shed more light on the molecular mechanisms of human CRC invasion. Importantly, because of the molecular differences, different treatment may be considered between EMT and crypt-like invasion subtypes. For example, a recent publication has elegantly shown that the efficacy of the PD-1/PD-L1 blockade therapy of several cancers is influenced by gut microbiome [52].

### 3.4. Dog-Human Comparison Could Be Effective for Driver-Passenger Discrimination for Missense Mutations

Driver-passenger discrimination has always been a central aim of cancer research. We have previously shown that our human-dog comparative genomics and oncology strategy is effective for driver-passenger discrimination for amplified/deleted genes in CRCs [7,10]. Our work here also indicates the potential of this approach on missense mutations. Indeed, known and putative drivers of *CTNNB1* and *TP53* are among the most frequent missense mutations detected in our canine tumors. The comparison can be expanded to numerous other genes that harbor one or multiple missense mutations, once the corresponding amino acid residues between the dog and human proteins are established.

Stromal drivers and microbial drivers are harder to identify, with fewer efficient approaches available. Our discovery of prominent fibroblast proliferation in canine invasive tumors, as well as significant enrichment of *H. bilis* and *A. finegoldii* in canine tumors may open a new avenue to address these important but difficult questions. Indeed, fibroblasts are known to play an important role in human CRC and other cancers [32,48,53], *H. bilis is* linked to human IBD and CRC [44], and *A. finegoldii* is detected in blood samples of human CRC patients [43].

Lastly, we acknowledge our current canine sample size is small. Because of the vast heterogeneity, a much larger sample size is required for efficient driver-passenger discrimination via dog-human comparison. Also note that our current study has relied on WGS of tumor samples for microbiome analysis, which may fail to detect less abundant bacterial species. Metagenomics data from stool samples should also be examined.

## 4. Materials and Methods

### 4.1. Canine Samples

Fresh-frozen (FF) canine tissues and spontaneous tumors were obtained from various veterinary colleges (Table S1). Samples were collected from client-owned dogs that develop the disease spontaneously, under the guidelines of the Institutional Animal Care and Use Committee for use of residual diagnostic specimens and with owner informed consent. The breed, age, histopathologic description and other information are provided in Table S1. The research received the ethical approval from the Institutional Animal Care and Use Committee (A2017 01-025-R1, approved on 8 February 2018 for University of Georgia; 2010A0015-R2, approved on 2 December 2017 for Ohio State University; and 16-6532A, approved on 22 March 2018 for Colorado State University).

### 4.2. Tissue Dissection, DNA and RNA Extraction, and Quality Control

Cryosectioning of FF tissues, H&E staining and cryomicrodissection were performed as described [9,10,11] to enrich tumor cells for tumor samples, and unaffected/normal epithelial cells for control/normal samples. Genomic DNA and RNA were extracted from the dissected tissues using the AllPrep DNA/RNA Mini Kit (cat. no. 80204) from QIAGEN (Germantown, MD, USA). Only samples with a 260/280 ratio of ~1.8 (DNA) or ~2.0 (RNA) and showing no degradation and other contaminations were subjected to further quality control with qPCR and qRT-PCR analysis with a panel of genes [9,10,11].

### 4.3. Immunohistochemical (IHC) Analysis

IHC experiments were performed with 5 μM tissue sections and with antibodies as described [9,11]. Images were taken with a Zeiss LSM 710 confocal microscope (ZEISS, Oberkochen, Germany).

### 4.4. Paired-End WGS and RNA-Seq

Illumina sequencing was conducted. Paired-end 125 × 125 bp WGS was performed in collaboration with the BGI-America and the High Throughput Genomics Core Facility at Huntsman Cancer Center at the University of Utah (Salt Lake City, UT, USA). RNA-seq was performed in collaboration with the Georgia Genomics Facility at the University of Georgia.

### 4.5. Sequence Data Analyses

Sequence data were analyzed following pipelines as described [8,9,11]. Briefly, WGS reads were aligned to the dog reference genome canFam3.1 with BWA v0.7.10 (bio-bwa.sourceforge.net). RNA-seq reads were mapped to the same reference genome using either TopHat 2.1.1 (ccb.jhu.edu/software/tophat/index.shtml) (for gene expression) or STAR v2.4.1c (github.com/alexdobin/STAR) (for mutation finding). Both RNA-seq-based canine gene annotation [11] and human xenoRefGene [9] annotation were used. Both WGS and RNA-seq reads were used for mutation discovery with GATK v3.6 and MuTect. Known canine single nucleotide polymorphisms (SNPs) were excluded as described [11]. WGS data were used to identify inversions/translocations and chimeric fusion genes [8,9,10,11]. For CNA discovery, correctly and uniquely mapped WGS read pairs were used [8,9,10,11]. Gene expression quantification with RNA-seq reads and other analyses were performed as described [8,9,11].

### 4.6. Microbiome Analysis

Microbiome analysis was performed as described [11]. Briefly, WGS and RNA-seq read pairs that could not be placed onto the canine genome were mapped with BWA v0.7.10 to two microbial genome databases: HMP (the reference genome database curated by the Human Microbiome Project) and ABG (all bacterial genomic sequences) [11]. The bacterial diversity was calculated *D* by:

(1) Simpson’s Diversity:
  $$D=1-\frac{\sum n_{i}\left(n_{i}-1\right)}{N\left(N-1\right)};$$
(2) Shannon-Wiener Diversity:
  $$D=-\sum p_{i\;}*\ln p_{i\;};\;p_{i\;}=n_{i}/N.$$

In both methods, $n_{i}$ is the total number of reads mapped to the $i^{\mathrm{th}}$ species, and *N* is the total number reads mapped to all species.

### 4.7. TCGA Data Analysis

RNA-seq expression and WGS data of TCGA human colon cancers were obtained from the NCI GDC data portal (portal.gdc.cancer.gov). The mutation data were downloaded from the cBioportal Cancer Genomics database (www.cbioportal.org). Subtyping was performed using ssGSEA enrichment scores of CRC signatures as summarized in Materials.

### 4.8. Data Access

Sequence data have been submitted to the NCBI SRA database with accession number PRJNA418842.

## 5. Conclusions

Consistent with our previous CNA study [10], our current findings support that dogs share the same CRC development and progression pathways as humans. Furthermore, our study sheds light on the molecular features unique to proliferative and invasive canine tumors. Importantly, we identified three modes of CRC cell invasion in dogs and humans. Our work reveals that CMS4 human colon cancers consist of two subtypes, EMT and crypt-like invasion, that differ in TGF-β signaling and microbe content.

## Supplementary Materials

The following are available online at http://www.mdpi.com/2072-6694/10/9/330/s1, Figure S1: Hierarchical clustering of 26 canine samples, Figure S2: GSEA of canine tumors with canonical CRC signatures and canine CTNNB1 mutations, Figure S3: Stromal signature gene expression and representative IHC images of canine crypt-like and EMT invasion, Figure S4: TP53 mutations in human CRCs from TCGA, Figure S5: Subtyping of human colon cancer from TCGA and mutation signatures, Table S1: Canine case information and clustering analysis, Table S2: Canine GSEA and gene mutation of canonical CRC pathways, Table S3. Gene expression of fibroblast activation markers, Table S4: Canine microbiome analyses, Table S5: TP53 mutations, Table S6: Human colon cancer subtyping and molecular features.
