# Supplementary material for: Proliferative and Invasive Colorectal Tumors in Pet Dogs Provide Unique Insights into Human Colorectal Cancer
Source: Cancers (Basel). 2018 Sep 14;10(9):330. doi: 10.3390/cancers10090330 (PMC6162437; doi:10.3390/cancers10090330)
Supplement: Supplementary file 1 [file cancers-10-00330-s001.zip › cancers-358916-supplementary.pdf]

Supplementary Materials: Proliferative and Invasive Colorectal Tumors in Pet Dogs Provide Unique Insights into Human Colorectal Cancer

Jin Wang, Tianfang Wang, Yanfang Sun, Yuan Feng, William C. Kisseberth, Carolyn J. Henry, Irene Mok, Susan E. Lana, Kevin Dobbin, Nicole Northrup, Elizabeth W. Howerth and Shaying Zhao

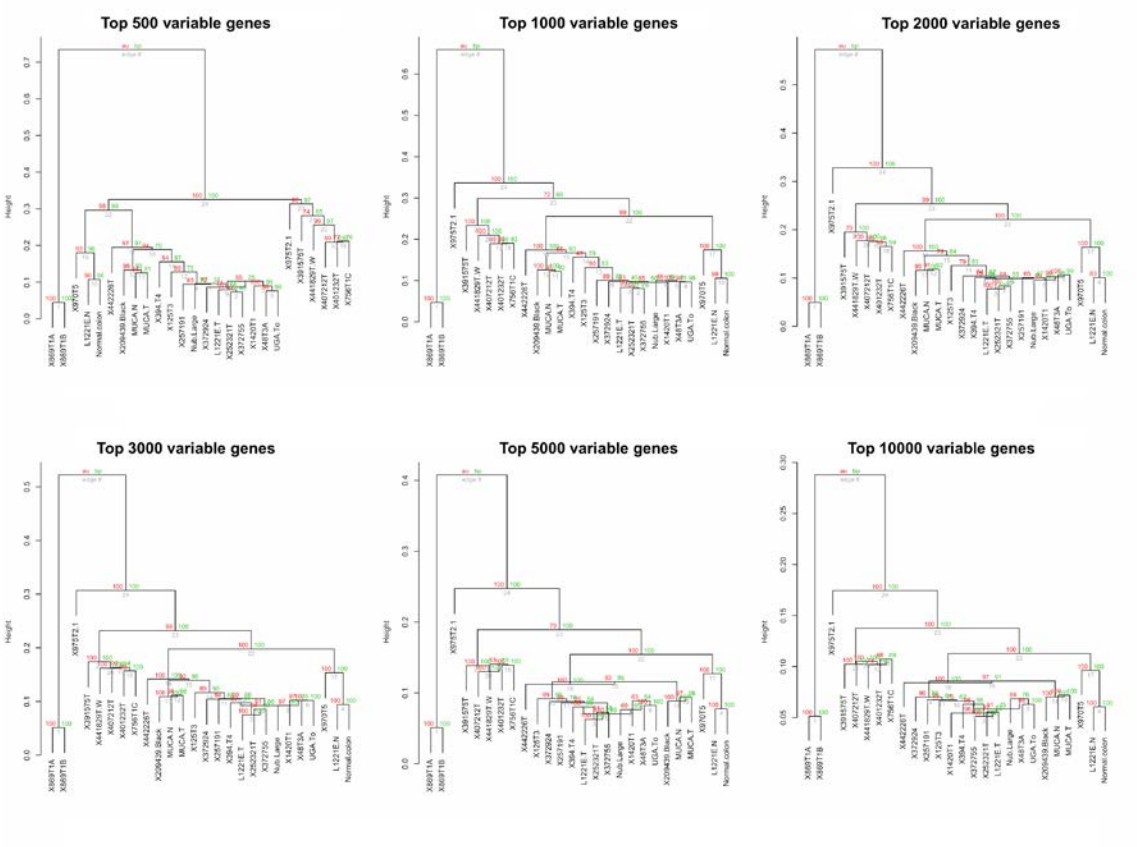

Figure S1. Hierarchical clustering, with various numbers of top most variable genes in expression across the 26 canine samples, consistently separates invasive tumors from proliferative tumors. Related to Figure 1B.

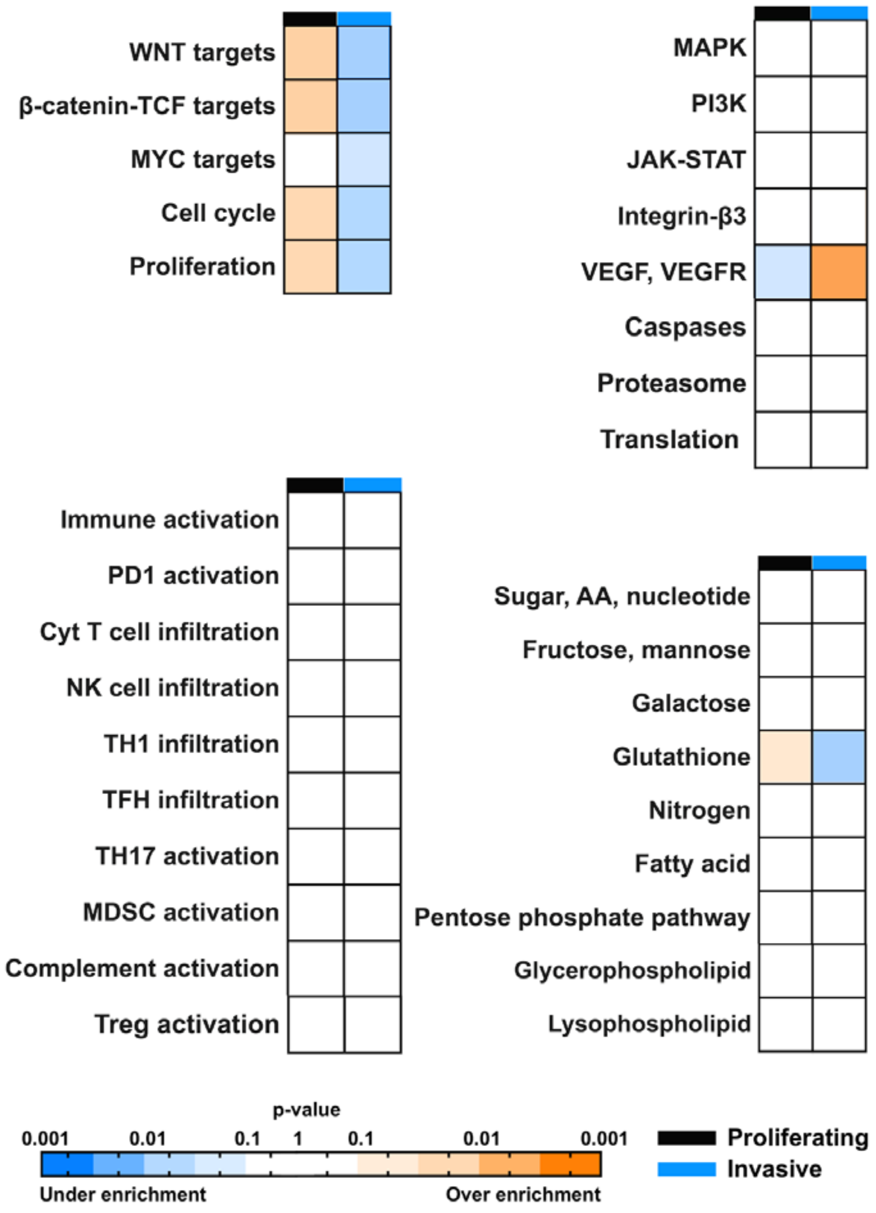

Figure S2A. The GSEA p-values between invasive and proliferative canine tumors with known human CRC pathway and sigature genes. Related to Figure 2A.

```
sp|B6V8E6|CTNB1_CANLF      MATQADLMELDMAMEPDRKAAVSHWQQQSYLDSGIHSGATTTAPSLSGKGNPEEEDVDTT
sp|P35222|CTNB1_HUMAN      MATQADLMELDMAMEPDRKAAVSHWQQQSYLDSGIHSGATTTAPSLSGKGNPEEEDVDTS
                           ***********************************************************:

sp|B6V8E6|CTNB1_CANLF      QVLYEWEQGFSQSFTQEQVADIDGQYAMTRAQRVRAAMFPETLDEGMQIPSTQFDAAHPT
sp|P35222|CTNB1_HUMAN      QVLYEWEQGFSQSFTQEQVADIDGQYAMTRAQRVRAAMFPETLDEGMQIPSTQFDAAHPT
                           ************************************************************

sp|B6V8E6|CTNB1_CANLF      NVQRLAEPSQMLKHAVVNLINYQDDAELATRAIPELTKLLNDEDQVVVNKAAVMVHQLSK
sp|P35222|CTNB1_HUMAN      NVQRLAEPSQMLKHAVVNLINYQDDAELATRAIPELTKLLNDEDQVVVNKAAVMVHQLSK
                           ************************************************************

sp|B6V8E6|CTNB1_CANLF      KEASRHAIMRSPQMVSAIVRTMQNTNDVETARCTAGTLHNLSHHREGLLAIFKSGGIPAL
sp|P35222|CTNB1_HUMAN      KEASRHAIMRSPQMVSAIVRTMQNTNDVETARCTAGTLHNLSHHREGLLAIFKSGGIPAL
                           ************************************************************

sp|B6V8E6|CTNB1_CANLF      VKMLGSPVDSVLFYAITTLHNLLLHQEGAKMAVRLAGGLQKMVALLNKTNVKFLAITTDC
sp|P35222|CTNB1_HUMAN      VKMLGSPVDSVLFYAITTLHNLLLHQEGAKMAVRLAGGLQKMVALLNKTNVKFLAITTDC
                           ************************************************************

sp|B6V8E6|CTNB1_CANLF      LQILAYGNQESKLIILASGGPQALVNIMRTYTYEKLLWTTSRVLKVLSVCSSNKPAIVEA
sp|P35222|CTNB1_HUMAN      LQILAYGNQESKLIILASGGPQALVNIMRTYTYEKLLWTTSRVLKVLSVCSSNKPAIVEA
                           ************************************************************

sp|B6V8E6|CTNB1_CANLF      GGMQALGLHLTDPSQRLVQNCLWTLRNLSDAATKQEGMEGLLGTLVQLLGSDDINVVTCA
sp|P35222|CTNB1_HUMAN      GGMQALGLHLTDPSQRLVQNCLWTLRNLSDAATKQEGMEGLLGTLVQLLGSDDINVVTCA
                           ************************************************************

sp|B6V8E6|CTNB1_CANLF      AGILSNLTCNNYKNKMMVCQVGGIEALVRTVLRAGDREDITEPAICALRHLTSRHQEAEM
sp|P35222|CTNB1_HUMAN      AGILSNLTCNNYKNKMMVCQVGGIEALVRTVLRAGDREDITEPAICALRHLTSRHQEAEM
                           ************************************************************

sp|B6V8E6|CTNB1_CANLF      AQNAVRLHYGLPVVVKLLHPPSHWPLIKATVGLIRNLALCPANHAPLREQGAIPRLVQLL
sp|P35222|CTNB1_HUMAN      AQNAVRLHYGLPVVVKLLHPPSHWPLIKATVGLIRNLALCPANHAPLREQGAIPRLVQLL
                           ************************************************************

sp|B6V8E6|CTNB1_CANLF      VRAHQDTQRRTSMGGTQQQFVEGVRMEEIVEGCTGALHILARDVHNRIVIRGLNTIPLFV
sp|P35222|CTNB1_HUMAN      VRAHQDTQRRTSMGGTQQQFVEGVRMEEIVEGCTGALHILARDVHNRIVIRGLNTIPLFV
                           ************************************************************

sp|B6V8E6|CTNB1_CANLF      QLLYSPIENIQRVAAGVLCELAQDKEAAEAIEAEGATAPLTELLHSRNEGVATYAAAVLF
sp|P35222|CTNB1_HUMAN      QLLYSPIENIQRVAAGVLCELAQDKEAAEAIEAEGATAPLTELLHSRNEGVATYAAAVLF
                           ************************************************************

sp|B6V8E6|CTNB1_CANLF      RMSEDKPQDYKKRLSVELTSSLFRTEPMAWNETADLGLDIGAQGEPLGYRQDDPSYRSFH
sp|P35222|CTNB1_HUMAN      RMSEDKPQDYKKRLSVELTSSLFRTEPMAWNETADLGLDIGAQGEPLGYRQDDPSYRSFH
                           ************************************************************

sp|B6V8E6|CTNB1_CANLF      SGGYGQDALGMDPMMEHEMGGHHPGADYPVDGLPDLGHAQDLMDGLPPGDSNQLAWFDTD
sp|P35222|CTNB1_HUMAN      SGGYGQDALGMDPMMEHEMGGHHPGADYPVDGLPDLGHAQDLMDGLPPGDSNQLAWFDTD
                           ************************************************************

sp|B6V8E6|CTNB1_CANLF      L
sp|P35222|CTNB1_HUMAN      L
                           *
```

Figure S2B. The protein alignment indicates that except for a T60S change, canine β-catenin is identical to human β-catenin. Related to Figure 2.

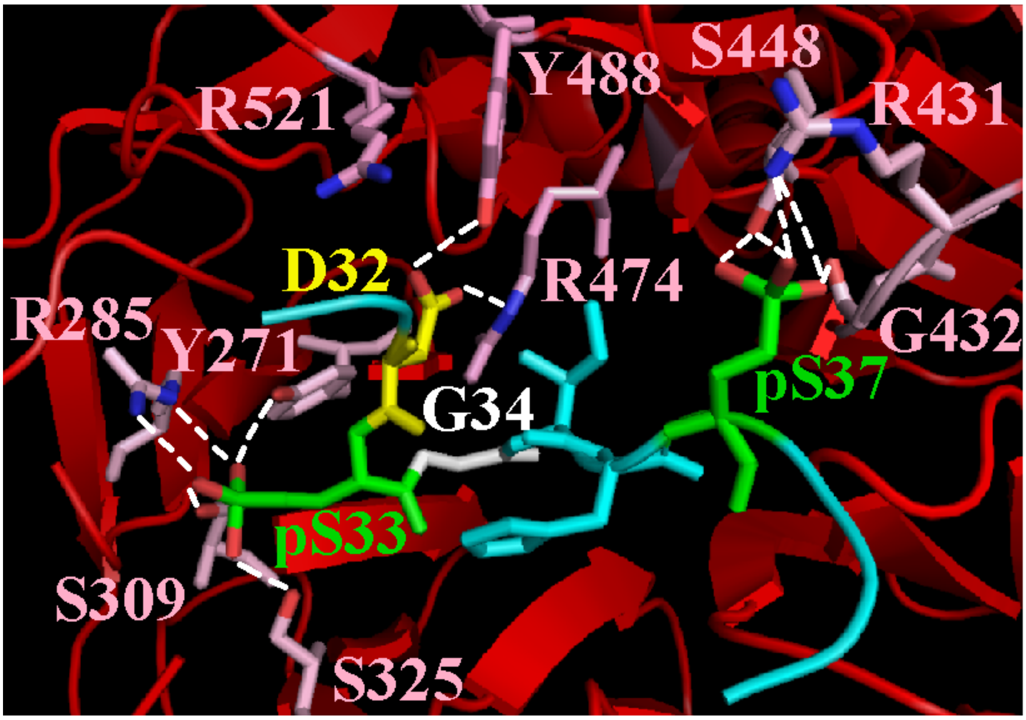

Figure S2C. The N-terminal phosphorylated peptide of β-catenin binds β-TrCP1 via hydrogen bonds and electrostatic interactions, based on a published human crystal structure (33). Related to Figure 2.

Figure S3A. Heatmaps indicate differential expression of signature genes for tumor microenvironment and for epithelial and mesenchymal features between proliferative and invasive tumors. Related to Figure 3.

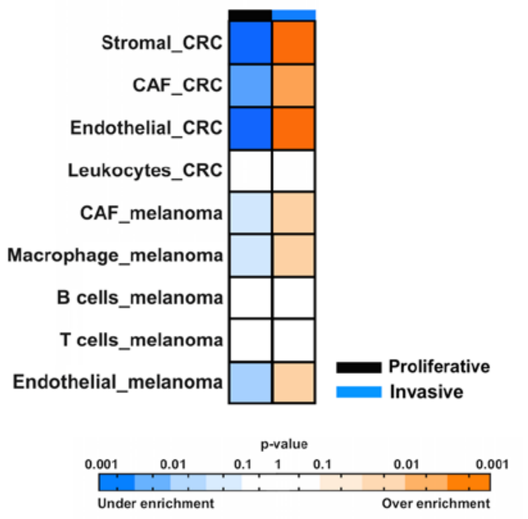

Figure S3B. GSEA p-values of under- or over-enrichment of signature genes for tumor microenvironment between proliferative and invasive canine tumors. Related to Figure 3.

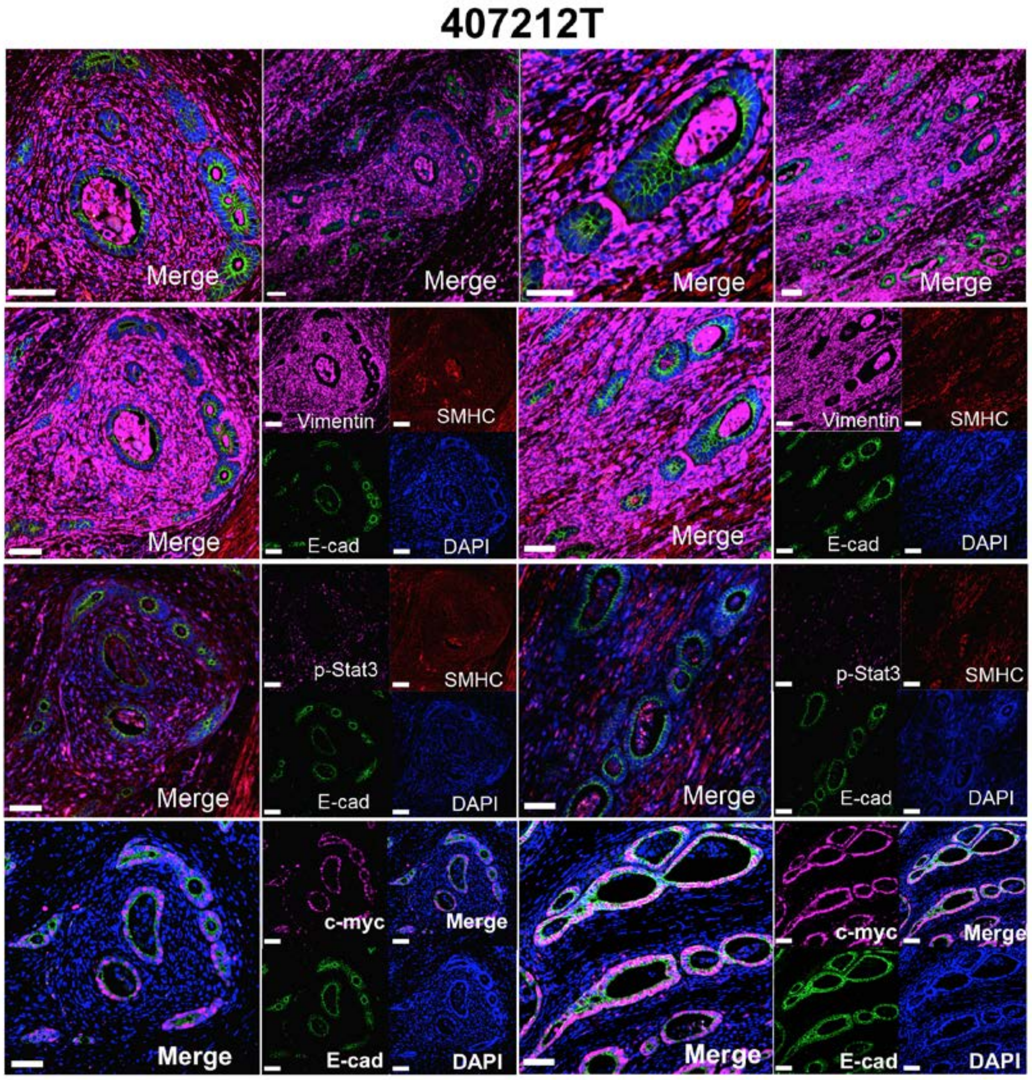

Figure S3C-1. Crypt-like invasion: with indicated marker staining. Related to Figure 3.

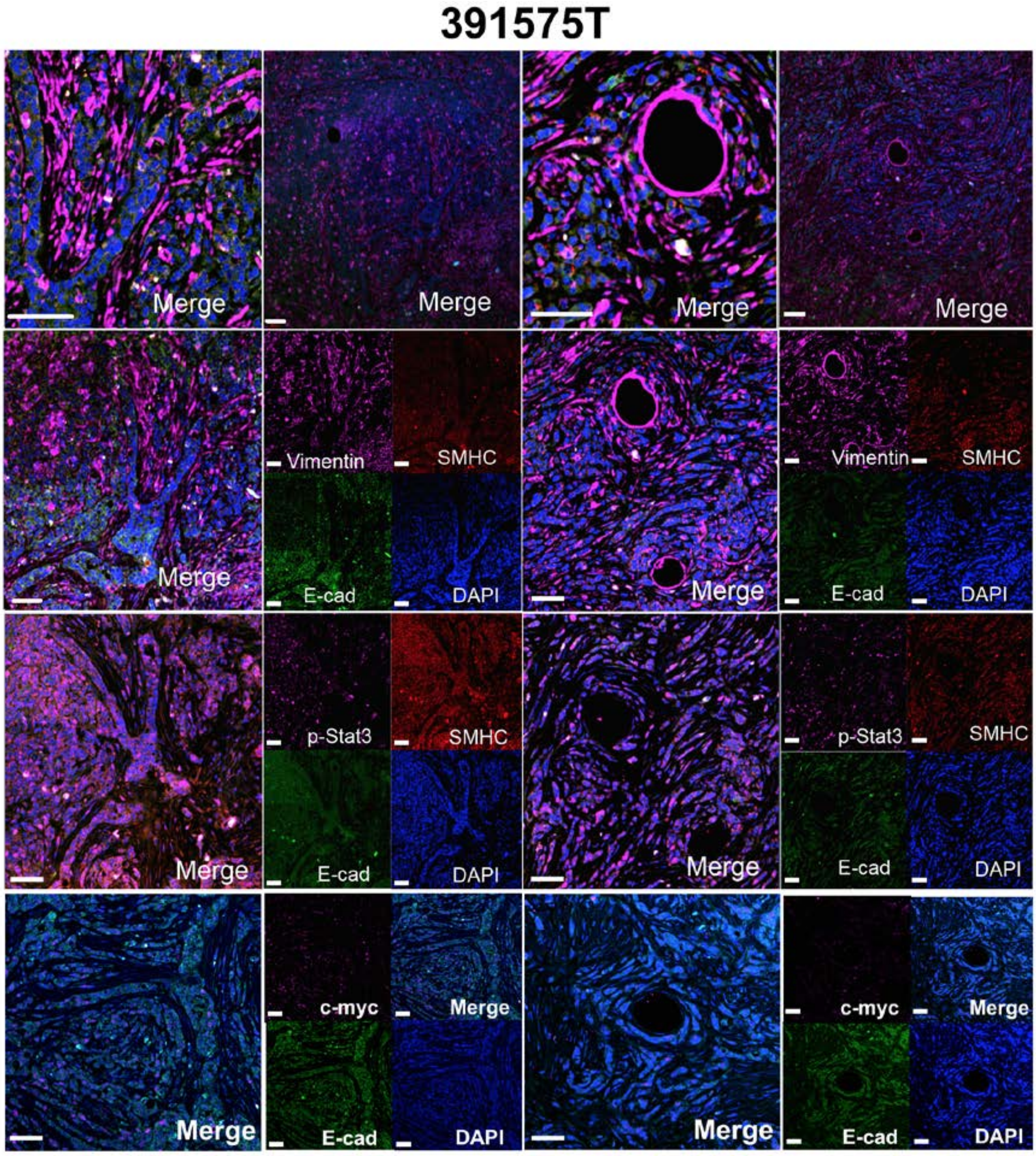

Figure S3C-2. EMT invasion: with indicated marker staining. Related to Figure 3.

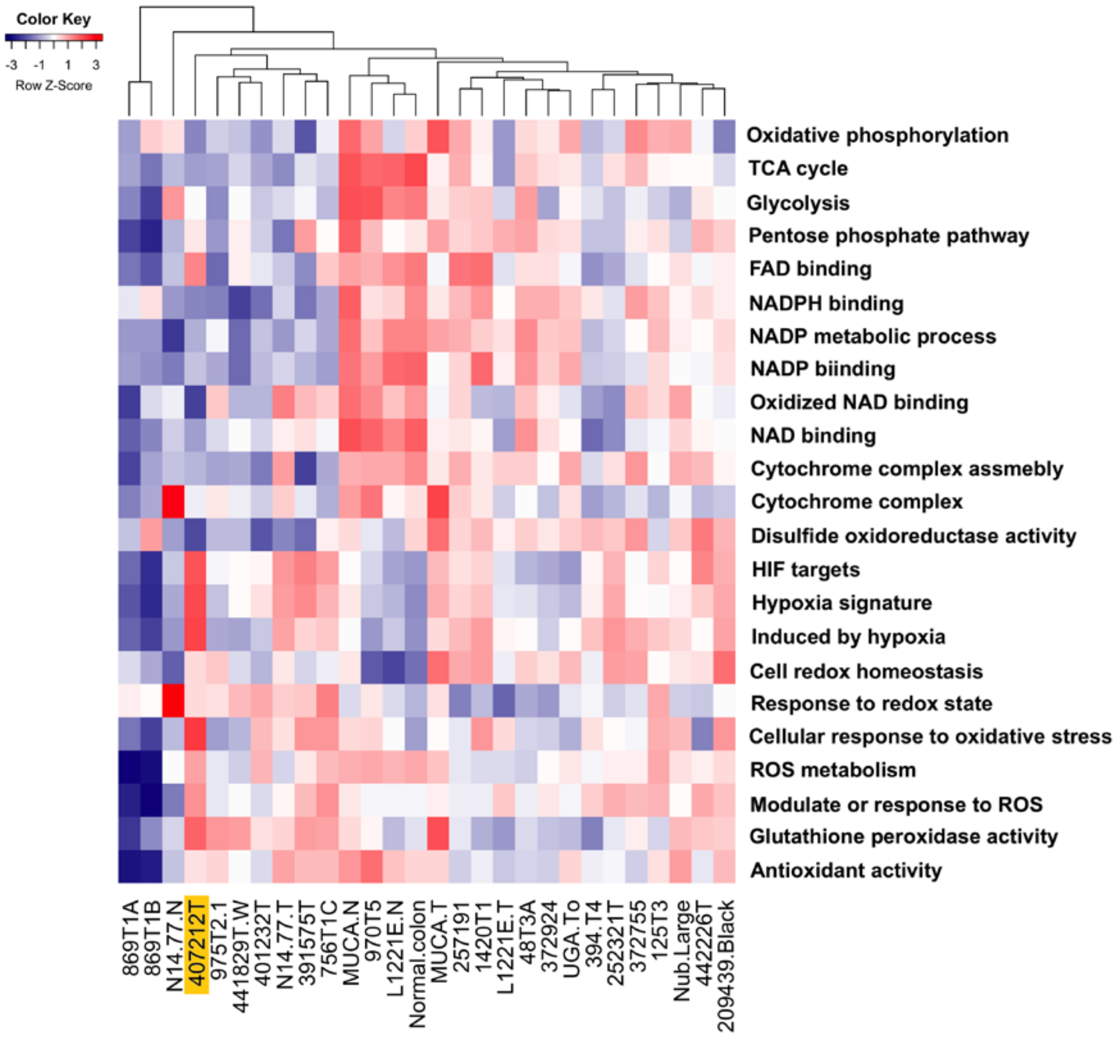

Figure S3D. Crypt-like invasion, as exemplified by the tumor highlighted in yellow, harbor activated signatures of hypoxia and cellular response to oxidative stress. Related to Figure 3.

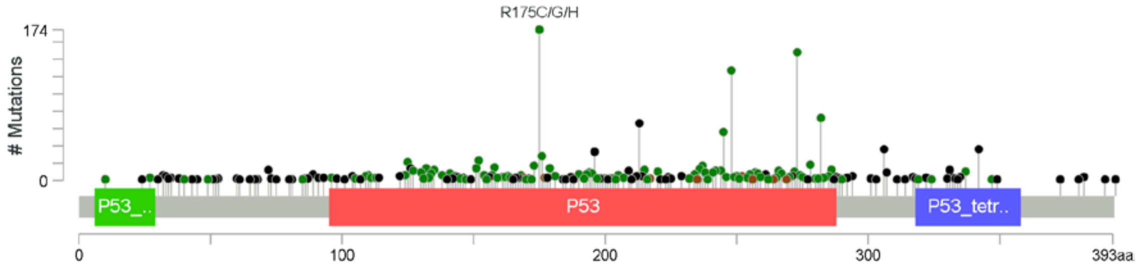

Figure S4. TP53 mutations in human CRCs from TCGA. The data are downloaded from the cBioprotal cancer genome database (http://www.cbioportal.org/). Related to Figure 5.

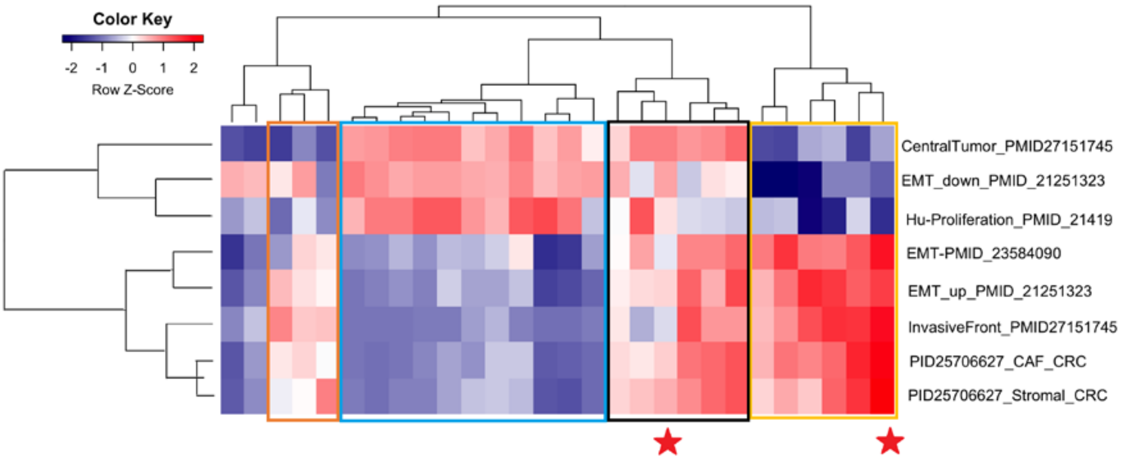

Figure S5A. Canine samples are clustered into five groups/subtypes: normal colon tissues (left), collective invasion (enclosed in orange box), proliferative (in blue box), crypt-like (in black box), EMT or stromal subtype (in yellow box), based on ssGSEA enrichment scores of published human CRC signatures shown. Red stars indicate the canine tumors that exemplify crypt-like invasion (left) and EMT invasion (right).

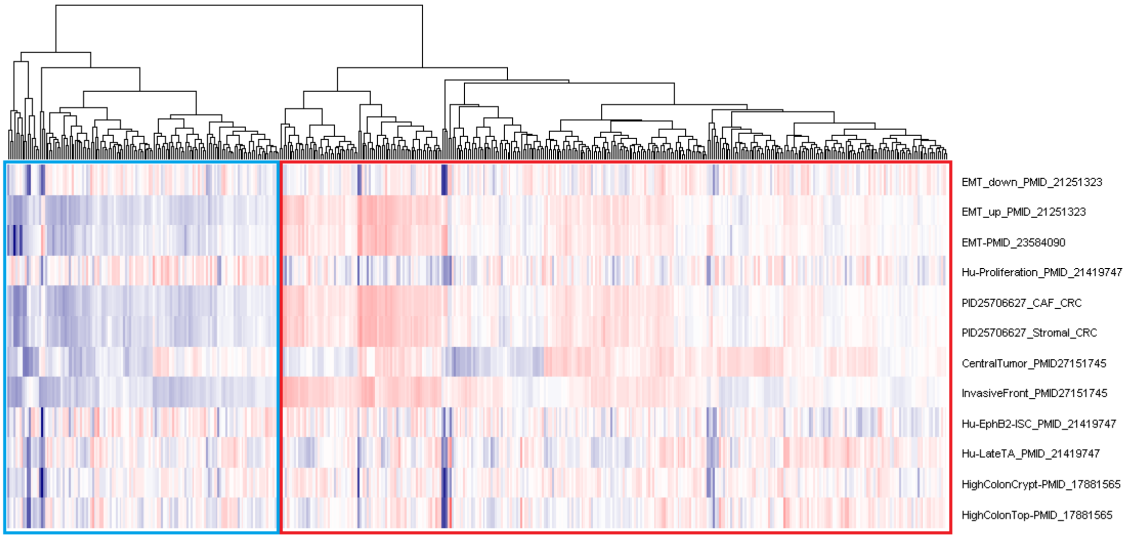

Figure S5B-1. Heatmap indicates that 478 human colon cancers of TCGA are clustered into two groups, proliferative (left, enclosed in blue box) and invasive (right, enclosed in red box), based on the ssGSEA enrichment scores of the 12 signatures indicated.

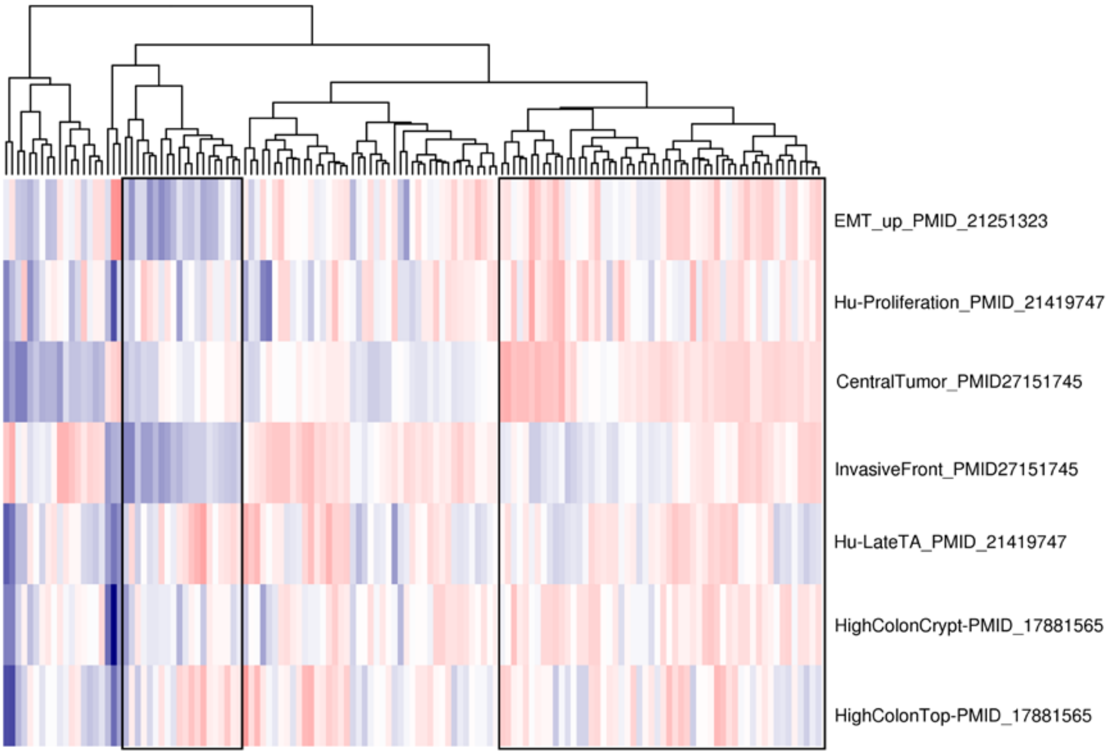

Figure S5B-2. Proliferative human colon cancers, enclosed in blue box in Figure S5B-1, are further clustered based on 7 signatures, selected based on ssGSEA enrichment score distributions among the samples. Clusters that are 1) enriched in central tumor and proliferation signatures and 2) depleted in invasive front signature, enclosed in black boxes, are classified as the primarily proliferative subtype shown in Figure 6A.

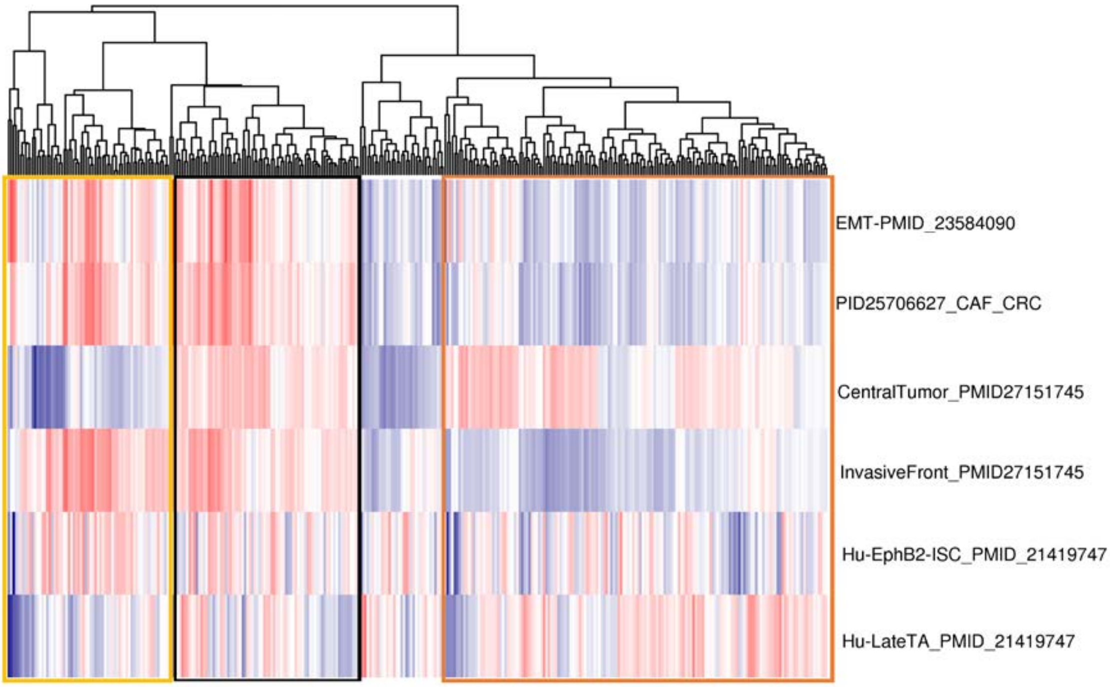

Figure S5B-3. Invasive human colon cancers, enclosed in red box in Figure S5B-1, are further clustered based on 6 signatures, selected based on ssGSEA enrichment score distributions among the samples. Three clusters are identified, including EMT (left, in yellow box), crypt-like (middle, in black box), and collective (right, in orange box).

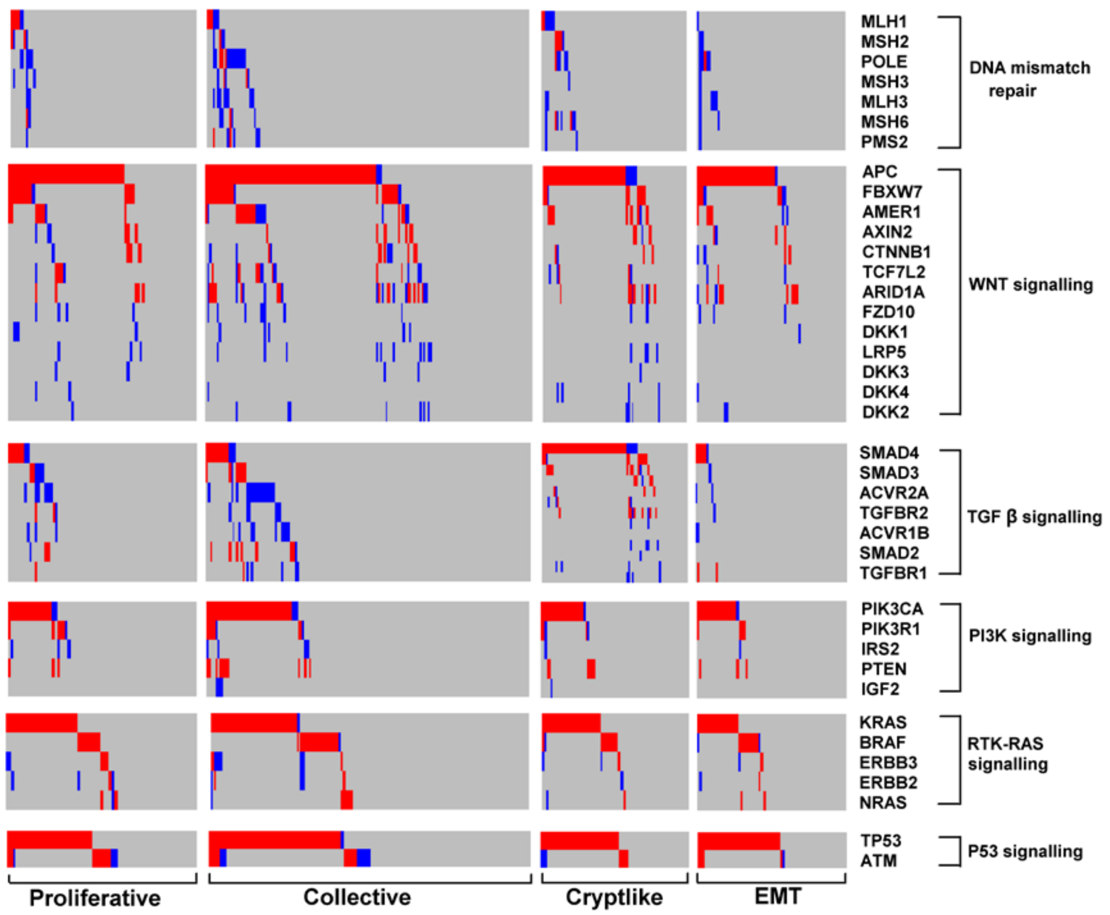

Figure S5C-1. Somatic driver (red) and passenger (blue) mutations of canonical pathway genes of human colon cancers of TCGA. Each column represents a tumor sample and each row represents a gene as shown. The data are from the cBioportal and detailed in Supplementary Table S6C-1.

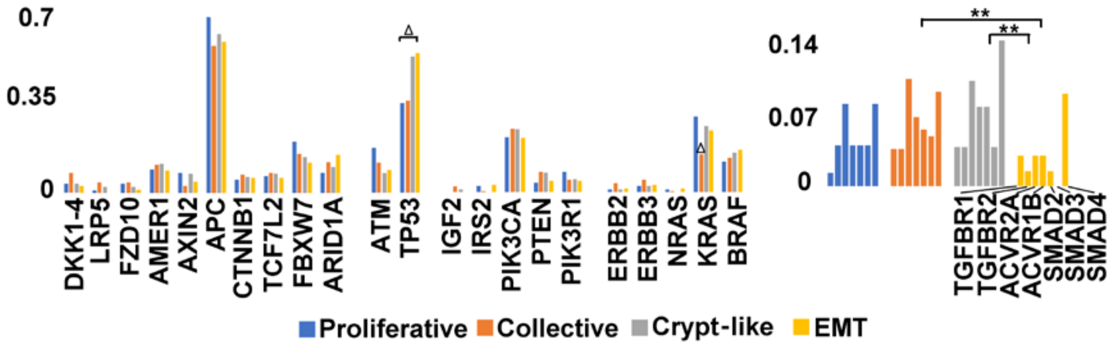

Figure S5C-2. The sample mutation rate of each gene in each human subtype shown in Figures 6C and S5C-1. The data are from the TCGA and detailed in Supplementary Table S6C-2.

© 2018 by the authors. Licensee MDPI, Basel, Switzerland. This article is an open access article distributed under the terms and conditions of the Creative Commons Attribution (CC BY) license (http://creativecommons.org/licenses/by/4.0/).
